# Change in the financial situation of students during COVID-19 and its impact on depressive symptoms: Sarah Negash

**DOI:** 10.1093/eurpub/ckac130.180

**Published:** 2022-10-25

**Authors:** S Negash, J Horn, E Heumann, SM Helmer, H Busse, K Heinrichs, CR Pischke, J Trümmler, J Niephaus, R Mikolajczyk

**Affiliations:** 1 Institute for Medical Epidemiology, Martin-Luther University Halle-Wittenberg, Halle (Saale), Germany; 2 Institute for Health and Nursing Science, Charité - Universitätsmedizin Berlin, Berlin, Germany; 3 Department Prevention and Evaluation, Institute for Prevention Research and Epidemiology, Bremen, Germany; 4 Institute of Medical Sociology, Heinrich Heine University Duesseldorf, Duesseldorf, Germany; 5 Department of Social Sciences, University of Siegen, Siegen, Germany

## Abstract

**Background:**

Students faced unique challenges during the COVID-19 pandemic that may have affected their financial situation as well as their mental health. This study sought to examine whether changes in financial situation before and during the COVID-19 pandemic were associated with depressive symptoms among German university students.

**Methods:**

The cross-sectional COVID-19 German Student Well-being Study (C19 GSWS; N = 7,267) was implemented at five German universities between 27.10. and 14.11.2021. Students were asked if they had had sufficient financial resources to cover monthly expenses before the pandemic, as well as during the first and third waves of the pandemic. Depressive symptoms were assessed using the CES-D 8 (score ranges 0-24) and the PHQ-2 (0-6); higher scores indicating more severe depressive symptoms. Linear regression models were used to examine associations between variables.

**Results:**

A worsened financial situation between the first and the third wave of the pandemic was associated with a one point (0.95) increase on the CES-D 8 scale (95% CI: 0.61, 1.29) and an improved financial situation with a decrease by 0.81 points (95% CI: -1.20, -0.42). A worsened financial situation was associated with a 0.26-point increase in PHQ-2 (95% CI: 0.14, 0.37) and an improved financial situation with a -0.12-point decrease (95% CI: -0.25, 0.01). Similarly, worsened financial situation in the third wave compared to prior to the pandemic, was also associated with an increase in CES-D 8 score and PHQ-2 and an improved financial situation with a decrease in CES-D 8 and PHQ-2.

**Conclusions:**

Our findings suggest associations between students’ financial situation during the COVID-19 pandemic and their mental health. Due to their instable financial situation, students are a vulnerable group in need of mental and financial support during pandemic crises.

**Key messages:**

- This study revealed associations between students’ financial situation and depressive symptoms.
- A comprehensive approach in efforts to address mental health, including financial support is required.

